# The Vulcanite Patent Case

**Published:** 1862-02

**Authors:** 


### THE VULCANITE PATENT CASE.

THE following decree was made by the United States Court for the Southern District of Ohio, in September last.

IN CHANCERY.—*Henry B. Goodyear*, vs. *John T. Toland.*

And now comes the said plaintiff, and it appearing to the Court that the said defendant, though ruled so to do, hath failed so to answer the said Bill of Complaint, the same is by the Court here ordered to be taken as confessed. And, therefore, the Court do find that the said two letters patent referred to in said Bill, being reissued patents Nos. 556 and 557 were duly granted and issued to said plaintiff as administrator of said Nelson Goodyear deceased, and that the same were and are in all respects valid, and secure to said plaintiff as such administrator the exclusive right of making, constructing, using, and vending to others to be used, the said improvements specified in said letters patent for the unexpired term of fourteen years, from the 6th of May, 1851. And the Court doth further find, that the said defendant has been engaged in manufacturing and selling large quantities of plates and bases for artificial teeth, and other articles made of the new substance described and secured by said letters patent, in violation of said exclusive right so secured to the plaintiff as aforesaid, and without the license or authority of the plaintiff. Whereupon, it is by the Court here ordered and decreed, that the said defendant be, and he is hereby enjoined and forbidden from henceforth, during the continuance of said letters patent, and during the term thereof yet unexpired, or any further term for which the same may be renewed or extended, from continuing the manufacture or sale of plates and gums for artificial teeth or other articles so made in violation of the said patents or either of them, and that the defendant pay the costs of this suit. And in as much as the Court is not now advised of the amount of damages which should be decreed to be paid by said defendant to the plaintiff on account of the said infringements and violations of the said patents, it is ordered that this case be referred to Thomas M. James, as Special Master, to state and return to this Court an account thereof, and this case is for that purpose further continued, and the said Special Master is authorized to call before him and examine under oath the said defendant, and such other witnesses as may be necessary in order to establish the extent of said damages.

United States of America, Southern District of Ohio, *ss.*

I, John McLean, Clerk of the Circuit Court of the United States, within and for the Seventh Circuit and Southern District of Ohio, do hereby certify the foregoing to be a true and accurate copy of a Decree in Chancery in the case of Henry B. Goodyear, administrator, etc., *vs.* John T. Toland, rendered on the 11th day of September, A. D. 1861, as the same remains upon the Journal of said Court in my office.

[L. S.] In testimony whereof I have hereunto set my hand and affixed the seal of said Court at Cincinnati, this 25th day of September, A. D. 1861, and in the 86th year of the Independence of the United States of America.

Attest: JOHN McLEAN, *Clerk, etc.*

This decision has been extensively circulated, and is pretty generally known by the profession; and it was supposed by many that this would be the conclusion of the matter, at least so far as this case was concerned.

It seems, however, by a recent turn to be quite otherwise, for the decree is set aside. Toland has filed his answer, and the case is again opened up, so that it now stands as it did before the decree was issued, at least so far as final adjustment is concerned.

In the answer filed by Toland, the following declarations are set forth :

First, That prior to the date of the patents, here declared to be infringed, many and various parties, both in this and other countries, vulcanized India Rubber, upon the same principle involved in this patent: also, that Nelson Goodyear was not the first and original inventor and discoverer of the theory, or any invention, or improvement patented, in and by said patent, or in and by either of said reissued patents, or of any material and substantial parts thereof. Also, that patents were granted, also, that the process was described and patented by various parties, both in this country and England, prior to the date of the patents here in question.

Second, That more is claimed, for these patents said to be infringed, than they embrace. That the original patent was admitted to be insufficient, that the reissues contained entirely new material, which it was claimed was not proper.

The defendant admits, that various suits for infringement, have been brought for infringement of these patents, but maintains, that in no case, has there been a fair test made of the real merits and validity of these patents.

It is further stated, that in 1852, letters patent were granted to John Rider, for improvement or discovery in the preparation and manufacture of Gutta Percha, with all the rights and privileges accruing thereto. That Henry B. Goodyear gave countenance to, and acquiesced in, the public sale and use of rights and privileges under said patents of Rider & Murphy, and that in legal effect, this amounts to an abandonment of the claims of their patents of 1851, at least so far as Gutta Percha is concerned. The defendant admits, that in his business for constructing artificial dentures, he used and vulcanized Gutta Percha, and India Rubber with a small quantity of sulphur, but not in proportion described in said reissued letters patent.

We understand the parties will begin very soon to take testimony. In regard to the merits of this case we do not pretend to be posted; we have never investigated it; both sides have said much, but this affords rather poor data for correct opinion.

The question involved is not one that, to any very great extent, affects the progress of the profession, either in its art or science.

Any improvement or discovery really valuable will sooner or later come into use, whether patented or not. In matters of this kind all should avail themselves of the advantages of the improvement, and in a legitimate way.

If the patent is valid and just, respect it, and proceed accordingly; if not, disregard it. The case under consideration we should be pleased to see decided by a fair contest. T.

